# Differential microRNA expression in the SH-SY5Y human cell model as potential biomarkers for Huntington’s disease

**DOI:** 10.3389/fncel.2024.1399742

**Published:** 2024-07-10

**Authors:** Ayaz Belkozhayev, Raigul Niyazova, Mohammad Amjad Kamal, Anatoliy Ivashchenko, Kamalidin Sharipov, Cornelia M. Wilson

**Affiliations:** ^1^Life Sciences Industry Liaison Lab, School of Psychology and Life Sciences, Canterbury Christ Church University, Sandwich, United Kingdom; ^2^M.A. Aitkhozhin Institute of Molecular Biology and Biochemistry, Almaty, Kazakhstan; ^3^Department of Chemical and Biochemical Engineering, Geology and Oil-Gas Business Institute Named after K. Turyssov, Satbayev University, Almaty, Kazakhstan; ^4^Faculty of Biology and Biotechnology, Al-Farabi Kazakh National University, Almaty, Kazakhstan; ^5^Novel Global Community Educational Foundation, Hebersham, NSW, Australia; ^6^Center for High Altitude Medicine, Institutes for Systems Genetics, West China School of Nursing, Frontiers Science Center for Disease-related Molecular Network, West China Hospital, Sichuan University, Chengdu, China; ^7^King Fahd Medical Research Center, King Abdulaziz University, Jeddah, Saudi Arabia; ^8^Department of Pharmacy, Faculty of Health and Life Sciences, Daffodil International University, Dhaka, Bangladesh; ^9^Centre for Global Health Research, Saveetha Medical College and Hospital, Saveetha Institute of Medical and Technical Sciences, Chennai, India; ^10^Enzymoics, Hebersham, NSW, Australia; ^11^Center for Bioinformatics and Nanomedicine, Almaty, Kazakhstan; ^12^Department of Biochemistry, Asfendiyarov Kazakh National Medical University, Almaty, Kazakhstan; ^13^University of Liverpool, Liverpool, United Kingdom

**Keywords:** biomarker, Huntington’s disease, miRNA, nucleotide repeats, target genes

## Abstract

Huntington’s disease (HD) is caused by an expansion of CAG trinucleotide repeat in the HTT gene; the exact pathogenesis of HD currently remains unclear. One of the promising directions in the study of HDs is to determine the molecular mechanism underlying the development and role of microRNAs (miRNAs). This study aimed to identify the profile of miRNAs in an HD human cell line model as diagnostic biomarkers for HD. To study HD, the human SH-SY5Y HD cell model is based on the expression of two different forms: pEGFP-Q23 and pEGFP-Q74 of HTT. The expression of Htt protein was confirmed using aggregation assays combined with immunofluorescence and Western blotting methods. miRNA levels were measured in SH-SY5Y neuronal cell model samples stably expressing Q23 and Q74 using the extraction-free HTG EdgeSeq protocol. A total of 2083 miRNAs were detected, and 354 (top 18 miRNAs) miRNAs were significantly differentially expressed (DE) (*p* < 0.05) in Q23 and Q74 cell lines. A majority of the miRNAs were downregulated in the HD cell model. Moreover, we revealed that six DE miRNAs target seven genes (*ATN1, GEMIN4, EFNA5*, *CSMD2, CREBBP, ATXN1,* and *B3GNT*) that play important roles in neurodegenerative disorders and showed significant expression differences in mutant Htt (Q74) when compared to wild-type Htt (Q23) using RT-qPCR (*p* < 0.05 and 0.01). We demonstrated the most important DE miRNA-mRNA profiles, interaction binding sites, and their related pathways in HD using experimental and bioinformatics methods. This will allow the development of novel diagnostic strategies and provide alternative therapeutic routes for treating HD.

## 1 Introduction

The expansion of CAG trinucleotide repeats located in the coding sequence (CDS) of various human genes is associated with the development of neurodegenerative disorders (NDs), including Huntington’s disease (HD) (Paulson, 2018; Ciosi et al., 2021). HD is an inherited pathology ND of the nervous system, which is caused by the abnormal expansion of a CAG trinucleotide repeat (>35 repeats) in the HTT gene on chromosome 4р16.3 (Belkozhayev A. M. et al., 2022). In patients, it is characterized by involuntary chorei movements, behavioral, and psychiatric disorders, including dementia (Roos, 2010). The normal “wild types” of the huntingtin protein play a synaptic function required in the postembryonic period, and it is considered to possess several cellular functions (Li and Wang, 2014). The mutation of expanded CAG results in cellular toxicity and can cause downregulation of diverse cellular responses, including proliferation, survival, and differentiation (Nucifora et al., 2001; Bates et al., 2006). An increasing number of studies have shown that mutant Htt proteins can affect negatively biomolecules, including microRNAs (miRNAs), in the HD brain (Fukuoka et al., 2018; Miniarikova et al., 2018; Tung et al., 2021).

The miRNAs are a class of small (18–25 nucleotides) non-coding RNA molecules. miRNAs are involved in biological processes, such as cell proliferation, apoptosis, and cell differentiation (Wang, 2010; Su et al., 2015) miRNAs are present in a stable form in easily accessible physiological fluids such as blood plasma, urine, and cerebrospinal fluid. In response to changes in the body, the level of certain miRNAs can significantly change various cellular processes (Ardekani and Naeini, 2010; Huang et al., 2011). Thus, miRNAs are an important family of new gene regulators, that are involved in various human diseases and could influence the response to various therapeutic treatments (Giza et al., 2014). The altered miRNA expression and the deregulation of genes controlled by miRNAs have been linked to many trinucleotides repeat diseases, including HD (Lee et al., 2011).

Neuronal cell lines and stem cell-derived models have been used to study miRNA dysregulation in HD. These models provided insights into how miRNAs may contribute to disease pathology at the cellular level. Previous studies have shown that 15 miRNAs are downregulated and 12 miRNAs are upregulated in a cell model for HD (Sinha et al., 2010). Other studies have demonstrated that animal models for HD revealed that numerous miRNAs displayed changes in their expression levels within the striatum and cerebral cortex. These changes predominantly led to a decrease in their expression (Martinez and Peplow, 2021). While there is proof of miRNA level changes in an HD model, these alterations were minor and lacked the necessary sensitivity to serve as a reliable biomarker. This study aimed to identify the profile of miRNAs in cell line models as diagnostic biomarkers for HD. Using the HTG EdgeSeq miRNA Whole Transcriptome Assay that measured the majority of known circulating miRNAs, the levels of 2083 miRNAs were determined in a human SH-SY5Y HD cell model based on the expression of Q23 (wt) and Q74 (mutant) of HTT.

## 2 Materials and methods

### 2.1 Cell culture and transfection

The SH-SY5Y human neuroblastoma cell line, which is widely used as a model for various ND such as Parkinson’s, Alzheimer’s, and HD, was obtained from the American Type Culture Collection (#ATCC/CRL-2266 ™, United States). Cells were maintained in growth medium Dulbecco’s modified eagle medium (DMEM) supplemented with 10% fetal bovine serum and 1% penicillin–streptomycin at 37°C in a humidified incubator (Thermo Scientific™, Heracell 150i Cell incubator, United States) with 5% CO_2_. The SH-SY5Y was transfected with pEGFP-Q23 and pEGFP-Q74 (Cat.No. #40261; #40262; Addgene, Watertown, MA, United States), which have normal and expanded Poly-Q tracts in *HTT*. Stable cell lines were created by using selection media DMEM containing 100 μg/mL of G418 (Sigma, UK).

### 2.2 Western blot analysis

For protein extraction, SH-SY5Y cells were rinsed with phosphate-buffered saline (PBS), and 0.25% trypsin/EDTA was used to detach cells from the surface. Protein lysates were prepared from cultured cells, and protein levels were quantified by DS-11 Series Spectrophotometer/Fluorometer (DENOVIX, United States). Approximately 50 μg of each sample was diluted with 2 x Laemmli’s loading buffer (60 mM Tris–HCl pH 6.8, 10% glycerol, 2% SDS, 50 mM dithiothreitol, and 0.01% bromophenol blue). The protein samples were run at 150 V for 80 min. The proteins were blotted onto an immobilon PVDF (Immobilon-P, transfer membrane Millipore, Sigma, Ireland, UK) membrane using a Trans-Blot Turbo Transfer System (Bio-Rad, United States) at 25 V for 60 min. The membranes were blocked with 5% non-fat dry milk (Marvel, Tesco, UK), diluted with TBST (10x Tris-buffered saline containing 0.1% Tween 20) on a tube roller for 1 h and then washed three times for 5 min in TBST. The membrane was incubated overnight at 4°C in a blocking solution with primary antibodies (1:500 mouse monoclonal anti-GFP (BD Bioscience, UK) or 1:2000 anti-mouse β-actin antibody (Sigma, UK) diluted in milk with TBST). Anti-β-actin was used as a loading control. The membrane was washed with TBST four times for 5 min and incubated with a secondary antibody for 1 h [goat anti-mouse HRP or goat anti-rabbit antibody HRP diluted in 5% milk with TBST (Sigma, UK)]. After washing, TBST Chemiluminescent Substrate (Thermo Fisher Scientific Inc., United States) was added to the membrane, and bands were visualized on ChemiDoc Imaging System (Bio-Rad Laboratories, Hercules, CA, United States).

### 2.3 Immunofluorescence

For immunofluorescence analysis, cells were seeded onto 16 mm coverslips at a density of 2 × 10^5^ cells well in 12-well culture plates for 24 h. The following day, the coverslips were washed three times with PBS and then fixed for 10 min with 100% methanol at −20°C. Then, the coverslips were incubated with PBS containing 0.5% bovine serum albumin (BSA) for 1 h at RT to block the non-specific binding of antibodies. Next, the coverslips were incubated in the diluted primary antibody (mouse monoclonal anti-α-tubulin; R&D Systems, MAB1195, dilution 1:500) in 1% BSA in PBS for 1 h at room temperature. The cells were then washed three times in PBS for 5 min each. Then, the cells were incubated with the secondary antibody (anti-mouse Alexa Fluor™ 647; 1:1000; Thermo Fisher, UK) in 1% BSA for 1 h at room temperature in the dark. The samples were washed three times again with PBS for 5 min each in the dark, followed by incubation with 30 nM DAPI (Sigma, UK), and washed a further three times with PBS for 5 min. Next, the coverslips were washed once with dH_2_0 prior to mounting in Mowiol-4-88. Images were obtained using the scanning disk immunofluorescent microscope (cellVivo, Olympus inverted microscope IX83. Tokyo, Japan).

### 2.4 HTG EdgeSeq miRNA whole transcriptome assay

The HTG EdgeSeq miRNA Whole Transcriptome Assay (miRNA WTA) (HTG Molecular Diagnostics, Tucson, United States) allows the measurement of the expression of 2083 human miRNA transcripts described in the miRBase database using next-generation sequencing (NGS). The test uses the HTG’s quantitative nuclease protection assay and utilizes the strong sensitivity and wide range of NGS. The HTG EdgeSeq device automates the nuclease protection phase, streamlining the library setup and making NGS platforms convenient for miRNA analysis. The chemistry, which only involves lysis and no extraction, notably cuts down the sample needed, unlike other techniques. The HTG EdgeSeq chemistry can be used with preserved tissue samples, biological fluids, and cultured cell lines. First, HTG lysis buffer (LB) was prewarmed for 20 min at 50°C. The cells were counted, and the pellets were washed with PBS prior to adding HTG LB. After 10 μL with a cell density of 4000 cells/μL, HTG LB was added, and the sample was stored at −80°C. Four aliquots of cells for each of the cell lines, Q23 and Q74, were submitted to HTG Molecular Diagnostics for analysis. Quantification of miRNA expression was performed using counts per million (CPM). To standardize and account for variations in total reads among samples, the CPM values were subjected to a log2 transformation.

### 2.5 miRNA-mRNA target analysis

The nucleotide sequences of DE miRNAs were downloaded from miRBase.^1^ The nucleotide sequences of the human mRNA genes were obtained from GenBank.^2^ The miRNA binding sites (BSs) were predicted by the MirTarget program. The MirTarget program defines the following features of binding: the start of the initiation of miRNA binding to mRNAs; the localization of miRNA BS in 5’UTRs, CDSs, and 3’UTRs; the free energy of binding; and the schemes of nucleotide interactions between miRNAs and mRNAs. The ΔGm equals the free energy of the miRNAs binding with their fully complementary nucleotide sequence. The MirTarget program finds hydrogen bonds between adenine (A) and uracil (U), guanine (G) and cytosine (C), G and U, and A and C. The distance between A and C was 1.04 nm, the distance between G–C and A–U was 1.03 nm, and the distance between G–U was 1.02 nm. For comparison, MirTarget differs from other (predicting miRNA target sites in mammalian mRNAs) programs in terms of finding the BSs of miRNA on the mRNAs in the following: (1) it accounts for the interaction of the miRNAs with mRNA over the entire miRNAs sequence; (2) it considers non-canonical G–U and A–C pairs; and (3) it calculates the free energy of the interaction of the miRNAs with mRNA (Ivashchenko et al., 2016; Belkozhayev A. et al., 2022).

### 2.6 Isolation of RNA

Total RNA was extracted from growing cell samples (>2 × 10^6^ to 5 × 10^6^) using the ReliaPrep RNA Cell Miniprep System (Promega, United States) following the manufacturer’s protocol. The concentration of RNA samples was quantified using a DS-11 series spectrophotometer. The total RNA samples were stored at −80°C until use.

### 2.7 cDNA and quantitative PCR

cDNA was prepared using the First Strand cDNA Synthesis Kit (Thermo Fisher, UK) following the manufacturer’s protocol. Approximately, 600 ng of RNA was prepared in a 10 μl sample. The samples were incubated using a PCR machine (3Prime, TECHNE. United States) at 25°C for 5 min, 37°C for 60 min, and 70°C for 5 min, and then the tubes were placed on ice, spun down in microfuge, and stored at −20°C for subsequent analysis. Single-strand DNA concentration was measured using a *DS-11 series spectrophotometer* and normalized to 10 ng/mL for the following experiments. The expression of different genes involved in NDs was quantified by Real-time PCR (qPCR) reactions performed with SYBR Green SuperMix (SsoAdvanced Universal SYBR^®^ Green Supermix; Bio-Rad Laboratories Ltd. UK), using 1 μL of cDNA template. A total reaction mixture was amplified in a 96-well PCR plate using the following standard thermocycling program: 95°C denaturation for 10 min, followed by 40 cycles of 95°C for 15 s, and 60°C for 30 s. All primer pairs were designed with Primer-BLAST^3^ and were obtained from Eurofins Genomics (Ebersberg, Germany) (Supplementary Table S1). The expression levels were measured in triplicate using the 2^−∆∆CT^ Ct (cycle threshold) method.

### 2.8 Statistical analysis

Normalization of miRNA expression data was performed using the EdgeSeq REVEAL^4^ from the HTG EdgeSeq System. The statistical results of RT-qPCR were analyzed by Student’s *t*-test, and a *p*-value of <0.05 was considered statistically significant. All gene fold change graph analyses were performed using the statistical software GraphPad Prism 9.

## 3 Results

### 3.1 Generation and expression of HTT (Q23 and Q74) in the SH-SY5Y cell line

**Figure 1:**
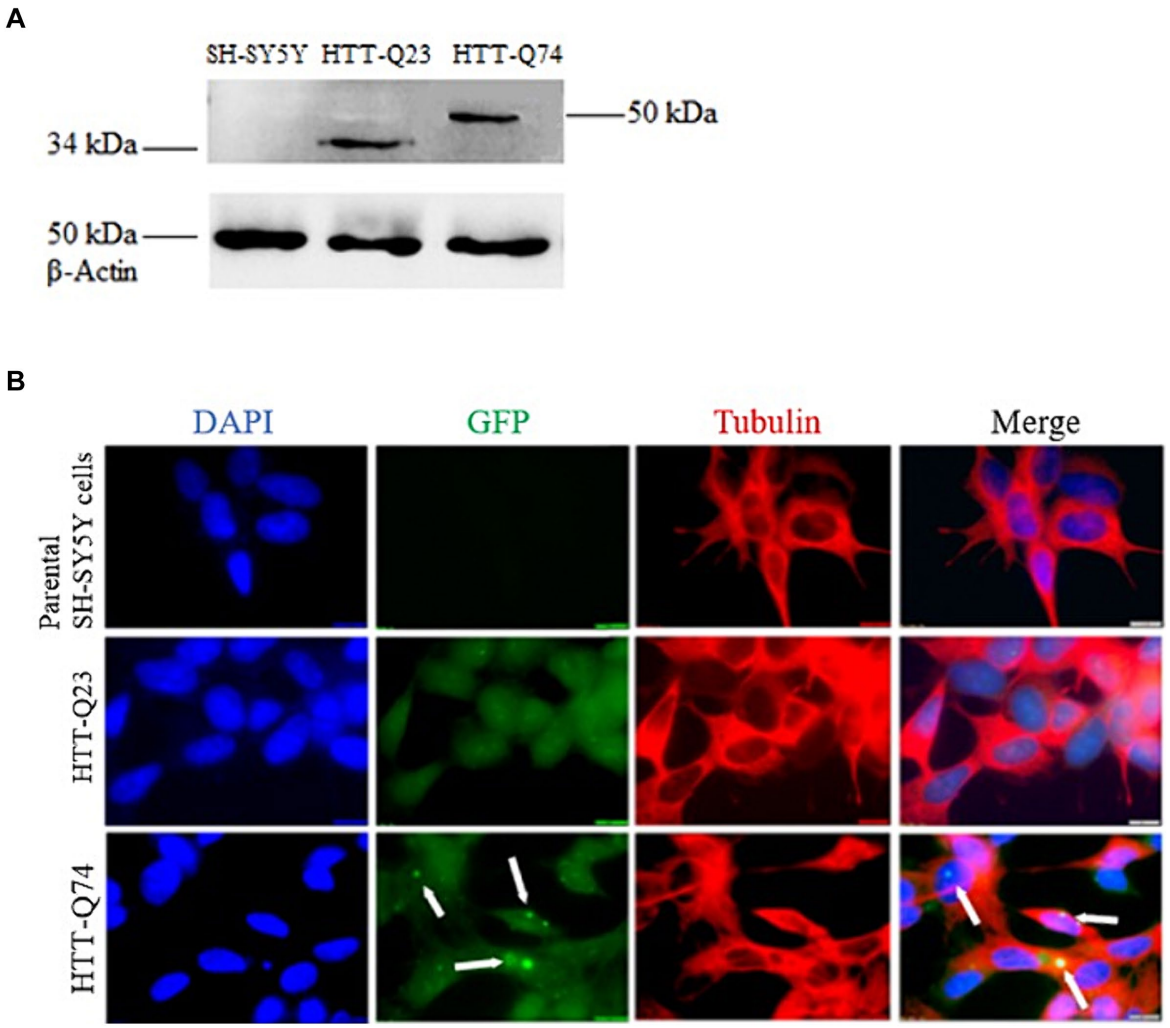
Western blot analysis of HTT-Q23/Q74 expression and immunofluorescence of non-transfected SH-SY5Y cells, HTT-Q23 and HTT-Q74 cell lines. **(A)** Western blot detection: no bands were observed in the parental SH-SY5Y cells. Protein products were observed in cell lysates from HTT-Q23 at 34 kDa and HTT-Q74 at 50 kDa, and β-actin was used as a protein loading control. **(B)** Immunofluorescence microscopy images: Cell nuclei were stained with DAPI. No GFP labeling was detected in the parental SH-SY5Y cells. The HTT-Q74 forms aggregates located predominantly in the nucleus, whereas HTT-Q23 was distributed evenly in the cytoplasm of the SH-SY5Y cells (magnification: 100 x; size bar = 10 μm).

The cellular model for HD was established by stably transfecting SH-SY5Y cells with vectors encoding wild-type Htt (Q23) and mutant Htt (Q74). As expected, band intensity of β-Actin expression was equal in all of the cell lines, demonstrating that there was equal loading of the protein samples. The wild-type Q23 migrated at approximately 34 kDa, while mutant Q74 migrated at 50 kDa, as it carries a larger CAG trinucleotide repeat (Figure 1A). The identification of components of Q74 aggregates containing Poly-Q proteins has proven difficult because of the insolubility of such complexes. Therefore, we dissolved it by utilizing acid and alkaline reagents previously demonstrated (Hazeki et al., 2000).

Immunofluorescence staining was performed on the parental SH-SY5Y and the stably transfected Q23-Htt and Q74-Htt cell lines. The results showed that the Q74-Htt protein was prone to aggregation compared to Q23. Furthermore, these results corroborated that Q74-Htt was localized in aggregates close to the nucleus, while Q23-Htt was evenly distributed in the cytoplasm (Figure 1B).

### 3.2 Differential analysis of miRNA expression in human cell models between HTT-Q23 and HTT-Q74 cell lines

In order to evaluate altered miRNA expression in SH-SY5Y cells stably expressing wild-type and mutant Htt, we performed differential expression of 2083 miRNA probes that passed expression filtering, quantified from small-RNA sequencing using the HTG EdgeSeq system. Profiles of wild-type Htt (Q23) and mutant Htt (Q74) were generated alongside multi-tissue control (MTC) control technical replicates using the HTG EdgeSeq system and all of them passed (Supplementary Table S2). Of the 2083 miRNAs, 354 miRNAs (top 18 miRNAs) were significantly differentially expressed (DE) in Q23 and Q74 cell lines, with 126 upregulated and 228 downregulated miRNAs (Supplementary Table S3).

From the DE 354 miRNAs, the top eight miRNAs (miR-3687, miR-4417, miR-1273 g-5p, miR-3648, miR-612, miR-1273 h-5p, miR-4484, and miR-5088-5p) were confirmed to have significantly increased in expression in HD. Moreover, 10 miRNAs (miR-411-5p, miR-32-5p, miR-301a-3p, miR-135a-5p, miR-876-3p, miR-889-3p, miR-148a-3p, miR-126-5p, miR-495-3p, and let-7f-2-3p) were downregulated in the Q23 and Q74 samples (Table 1).

**Table 1:** Top 18 differentially expressed miRNAs between wild type Htt (Q23) and mutant Htt (Q74) (upregulated miRNAs are in blue and downregulated miRNAs are in red).

miRNA	Mean normalized control	Mean normalized disease	Mean expression	Fold change	adjP disease.vs.control
miR-3687	10,239	40,520	14.63	3.96	2.7199
miR-4417	2,586	6,998	12.23	2.71	4.0280
miR-1273 g-5p	253	714	8.92	2.82	0.0008
miR-3648	198	458	8.36	2.30	0.0008
miR-612	84	268	7.46	3.14	0.0011
miR-1273 h-5p	2,594	5,753	12.03	2.22	0.0017
miR-4484	243	497	8.53	2.04	0.0028
miR-5088-5p	73	188	7.02	2.57	0.0036
miR-411-5p	46	3	4.61	−17.69	0.0007
miR-32-5p	646	56	8.46	−11.61	0.0008
miR-301a-3p	2,813	224	10.57	−12.57	0.0008
miR-135a-5p	49	6	4.80	−8.08	0.0011
miR-876-3p	130	16	6.19	−8.05	0.0012
miR-889-3p	34	3	4.18	−14.05	00013
miR-148a-3p	973	88	9.05	−11.07	0.0013
miR-126-5p	434	37	7.88	−11.79	0.0014
miR-495-3p	281	30	7.28	−9.39	0.0014
let-7f-2-3p	38	5	4.43	−7.08	0.0014

**Figure 2:**
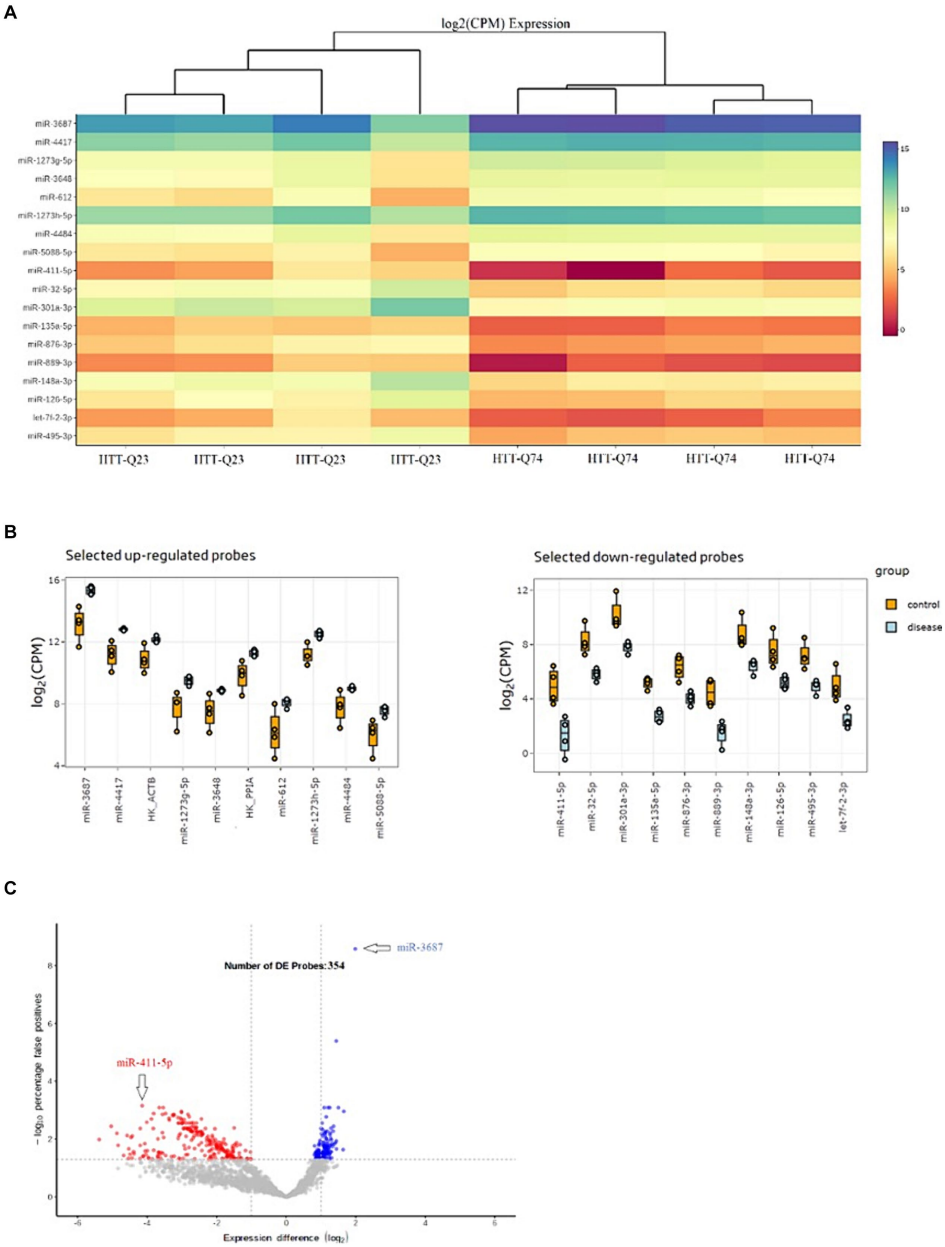
Differential Expression Results of miRNAs. **(A)** Heat map of the top 18 miRNAs that were differentially expressed in HTT-Q74 and HTT-Q23 cell lines. The color shown illustrates the relative expression level of the indicated miRNA across all samples (blue upregulated, red downregulated miRNAs). **(B)** Upregulation and downregulation of miRNA expression in disease (HTT-Q74) compared with the control (HTT-Q23). **(C)** Volcano plot showing the significantly differentially (*p* < 0.05) expressed 354 miRNAs in normal and mutant Htt groups.

Expression profiles of the 18 most significant miRNA heatmaps are depicted in Figure 2A. These 18 miRNAs were significantly dysregulated among the normal and mutant Htt groups (Figure 2B). As depicted in Figure 2C, the volcano plot shows sample clustering based on the expression of the 354 significant miRNAs. The wild-type Htt (Q23) and mutant Htt (Q74) groups clustered in distinct sets based on this group of miRNAs.

### 3.3 *In silico* analysis of the interaction of differentially expressed miRNAs with human mRNA genes

The characteristics of the interaction of DE 126 upregulated and 228 downregulated miRNAs with the mRNA of 17,494 human genes were studied using the MirTarget program. It was found that the differentially upregulated 126 miRNAs have 52,911 BSs on 8,482 target mRNAs with ΔG/ΔGm values ranging from 80 to 100% in 3’UTRs, 5’UTRs, and CDSs. Moreover, thirteen 412 BSs were also identified for 228 downregulated miRNAs in the 3’UTRs, 5’UTRs, and CDSs mRNA of 6,618 genes with ΔG/ΔGm values equal to 80 to 100%.

The obtained characteristics of the BSs of differentially upregulated and downregulated miRNAs (miR-3687, miR-612, miR-4417, miR-4261, miR-504-3p, miR-126-5p, miR-411-5p, miR-889-3p, and miR-22-5p) were identified in the 3’UTRs, 5’UTRs, and CDSs mRNAs of 18 genes, which play an important role in NDs, including HD. The list of target gene associations with neurodevelopmental disorders is mined from a weekly updated web resource of DISEASES^5^ and PubMed,^6^ (Table 2).

**Table 2:** Characteristics of differentially expressed miRNA interaction with genes mRNAs and association with neurodegenerative diseases.

**miRNA**	**Gene**	Start of BS, nt	ΔG, kJ/mol	ΔG/ΔGm, %	Region	Length, nt	Disease [PMID]
miR-3687	*B3GNT2*	77	−113	82	5’UTR	24	Autoimmune disease [35079000], [34790667]; [16513876]; schizophrenia [23904455]
miR-612	*CSMD2*	4,047	−119	85	CDS	25	Neuropsychiatric disease, schizophrenia, autism spectrum disorder [31068362]
	*GNAO1*	1958	−117	83	CDS	25	ND with involuntary movements [35782616]
	*ATXN2*	2,810	−114	82	CDS	25	ND, Spinocerebellar ataxia 2 [36511898]
miR-4417	*DUSP15*	950	−98	92	CDS	18	Autistic disorder [34257739]; Schuurs–Hoeijmakers Syndrome [28628100]; Brain disease [35082514]
	*EPN3*	1762	−96	90	CDS	18	Autosomal dominant non-syndromic deafness [33842346]
	*GLIS1*	405	−96	90	5’UTR	18	ND [32699115], [29779043]; Parkinson’s Disease [22759478]
	*CREBBP*	6,004	−87	82	CDS	18	ND (Alzheimer’s disease) [36441024]; Brain disease (associated with HTT gene) [36246562], [36466848], [20448484]
miR-4261	*ATN1*	1758÷2,319 (2)	−72÷ −76	83÷88	CDS	16	ND, trinucleotide repeat disorders, Dentatorubral-pallidoluysian atrophy, Spino. Ataxia Type 7 [36176563], [35401096], [35295941], [8136840]
	*GLIS1*	1928	−74	85	CDS	16	ND [32699115], [29779043]; Parkinson’s Disease [22759478]
	*CREBBP*	6,351	−70	80	CDS	16	ND (Alzheimer’s disease) [36441024]; Brain disease (associated with HTT gene) [36246562], [36466848], [20448484]
	*HAP1*	180÷3,147(2)	−70	80	CDS÷3UTR	16	HD [36509909], [36251080]; Brain disease [35609730], [35327413]
miR-504-3p	*ATXN1*	1,230	−96	80	CDS	21	Spinocerebellar ataxia type 1 [37238658]
miR-126-5p	*DNM1L*	2,154	−87	82	CDS	21	ND [34307245], [36302323], [35916724]
	*GEMIN4*	2,162	−91	86	CDS	21	ND, Neuromuscular disease [35295849], [34984269]
	*EFNA5*	369	−89	81	CDS	21	ND [5406389], [35105727], [35321205]
miR-411-5p	*NOS3*	1,040	−89	81	CDS	21	Alzheimer’s disease [16813604], [11041283], [18183499], [12697290]
	*EFNA5*	369	−89	81	CDS	21	ND [5406389], [35105727], [35321205]
miR-889-3p	*CNPY1*	796	−85	82	3’UTR	21	ND [34638438], [34314101], [32570916]
	*KIAA1324L*	1,633	−85	82	CDS	21	Predicted to be involved in negative regulation of nervous system development [17475571], [21177533]
miR-22-5p	*ATXN10*	1,126	−93	83	CDS	22	Spinocerebellar Ataxia Type 10 [36199580], [36092952], [35469073]

NDs, neurodegenerative diseases; HD, Huntington’s disease. In parentheses, () indicate the number of miRNAs BSs.

miR-3687 binds to the mRNA of the *B3GNT2* gene with −113 kJ/mole free binding energy in the 5′UTR. Furthermore, the BSs of miR-612 in CDS mRNAs of the *CSMD2, GNAO1,* and *ATXN2* genes show the highest free binding energy, equal to −114 and − 119 kJ/mole. miR-4417 and miR-4261 are bound in mRNAs of the *DUSP15, EPN3, GLIS1, CREBBP*, *ATN1,* and *HAP1* genes, with free binding energy changing from −70 kJ/mole to −98 kJ/mole in the 3’UTRs, 5’UTRs, and CDSs. The mRNAs of some genes have BSs for miR-4261 within their 3′UTRs and CDSs. For example, the 3′UTRs and CDSs of the *HAP1* gene have miR-4261 BSs. Furthermore, miR-4261 had two BSs in the mRNA of the *ATN* and *HAP1* genes, whose BSs started at 1758÷2,319 nt and 180÷3,147 nt with a length of 16 nt for 3′UTRs and CDS. The *ATXN1* gene contains BSs for miR-504-3p in the CDS with −96 kJ/mole free energy.

Downregulated miR-126-5p, miR-411-5p, miR-889-3p, and miR-22-5p bind to the mRNA of the *DNM1L, GEMIN4, EFNA5, NOS3, CNPY1, KIAA1324L,* and *ATXN10* genes in the UTRs and CDSs. The free energy values of these miRNA interactions with the target genes were equal to −88 ± 5 kJ/mole. According to Table 2, among the genes associated with NDs, the *HAP*1 and *CREBBP* genes play an important role in HD (Jiang et al., 2006; Li et al., 2019). In addition, the *ATXN2, ATN1, ATXN1,* and *ATXN10* genes belong to a group of genes that are associated with microsatellite-expansion diseases, a class of neurological and neuromuscular disorders caused by the expansion of short stretches of repetitive DNA, such as spinocerebellar and cerebellar ataxia (Suzuki and Yazawa, 2011; Ashizawa, 2012; Laffita-Mesa et al., 2021; Chen J. M. et al., 2022).

**Figure 3:**
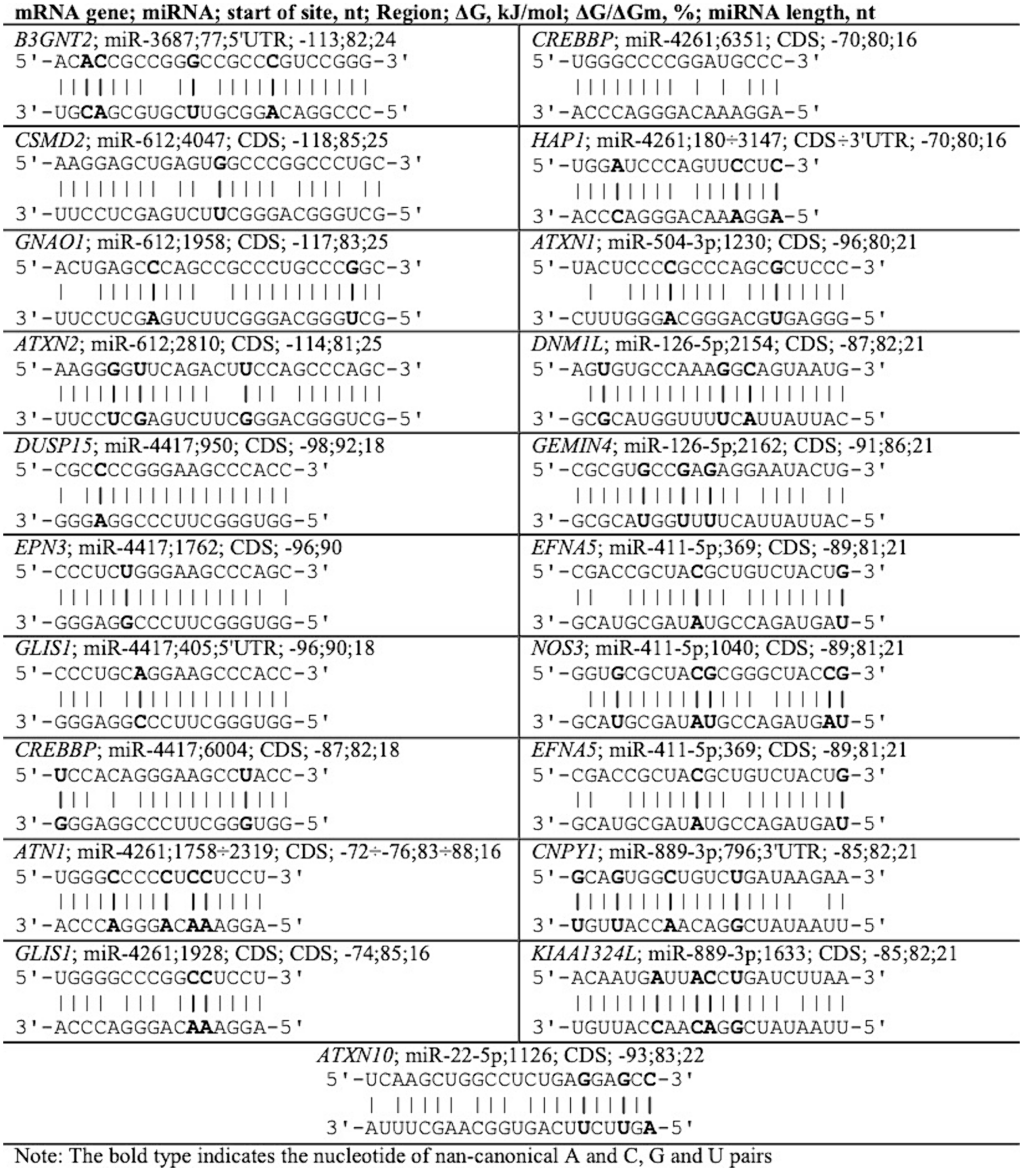
Schemes of miRNA interaction with mRNA of genes associated with neurodegenerative diseases.

The schemes of interaction of miR-3687, miR-612, miR-4417, miR-4261, miR-504-3p, miR-126-5p, miR-411-5p, miR-889-3p, and miR-22-5p with mRNAs of *B3GNT2, CSMD2, GNAO1, ATXN2, DUSP15, EPN3, GLIS1, CREBBP, ATN1, HAP1, ATXN1, DNM1L, GEMIN4, EFNA5, NOS3, CNPY1, KIAA1324L,* and *ATXN10* genes are shown in Figure 3. The schemes of the interaction of miRNAs with the mRNA of genes indicate the complementarity of nucleotides, including canonical (A–U, G–C) and non-canonical (A–C, G–U) bonds.

*In silico* analysis of DE miRNAs and their interactions with human mRNA genes, which play an important role in NDs, offers a valuable approach to deciphering intricate gene regulatory networks. This computational method provides insights into potential miRNA-mediated regulatory mechanisms and their implications for various biological processes and diseases. The integration of computational predictions with experimental validation and functional enrichment analysis contributes to our understanding of the complex interplay between miRNAs and their target genes, and these results show that miRNAs can regulate the expression of genes associated with the development of ND, including HD. Therefore, we also determined the expression of target genes by RT-qPCR to obtain a more accurate result for the prediction.

### 3.4 Analysis of differentially expressed genes

Next, the genes identified by a combination of miRNA profiling of Q23/Q74 cell line models with bioinformatic analysis were selected for validation by RT-qPCR analysis. To study the effect of Q74 on the gene expression of the selected genes, 18S was used as a housekeeping gene.

There was no statistical difference between the *GNAO1, ATXN2, DUSP15, EPN3, GLIS1, HAP1, DNM1L, NOS3, CNPY, KIAA1324,* and *ATXN10* genes. The study of 18 genes identified 3 genes to be upregulated and 4 genes to be downregulated.

**Figure 4:**
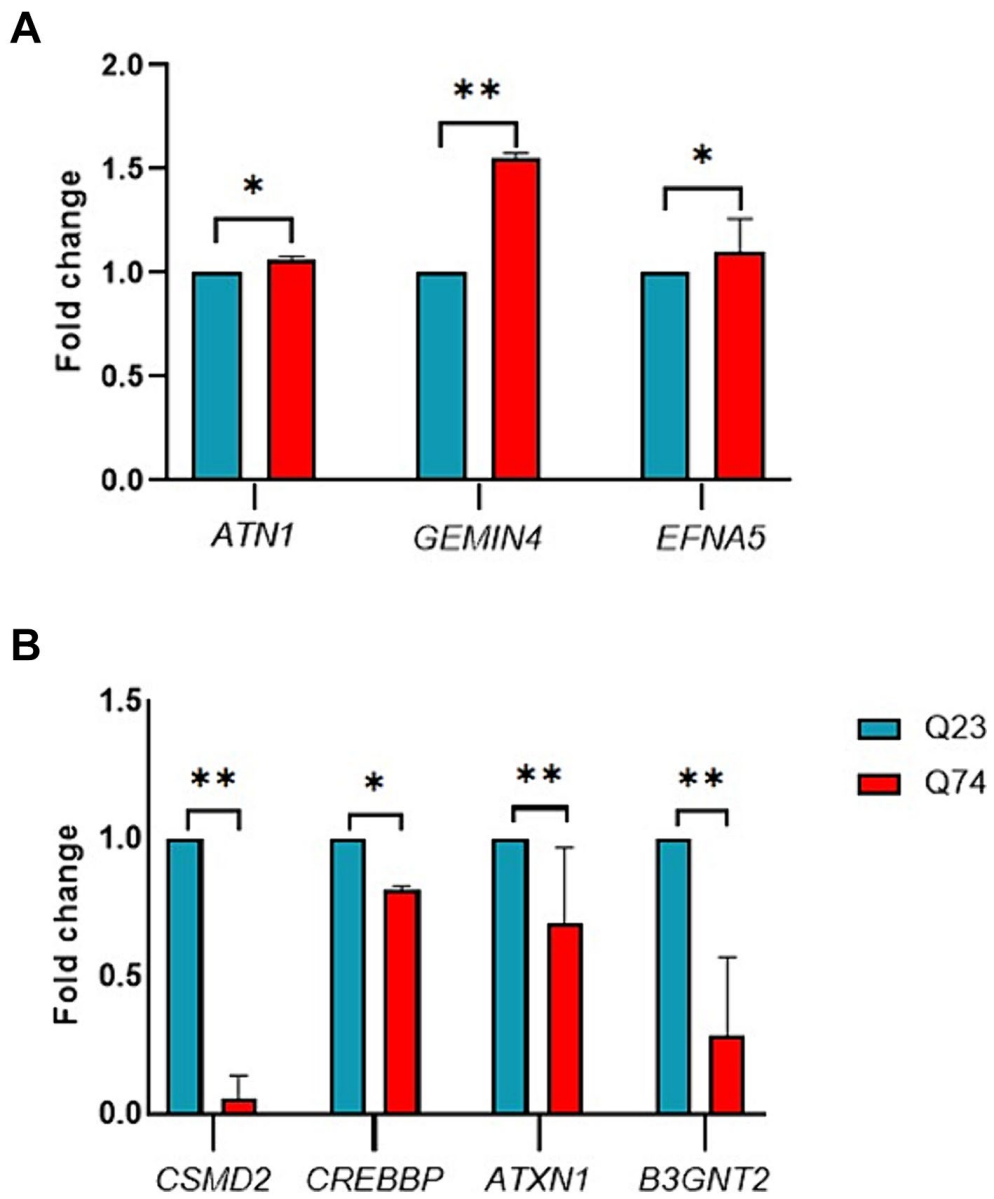
RT-qPCR validation of candidate differentially expressed genes between wild (Q23) type and mutant (Q74). **(A)** Relative expression of upregulated ATN1, GEMIN4, and EFNA5 genes. **(B)** Relative expression of downregulated CSMD2, CREBBP, ATXN1, and B3GNT2 genes (* and ** indicate *p* < 0.05 and 0.01, respectively).

The upregulated genes were *ATN1, GEMIN4,* and *EFNA5* (Figure 4A). From those genes upregulated, expression was the highest for *GEMIN4* 1.5-fold (*p* = 0.0022), while marginal upregulation was observed for *ATN1* (*p* = 0.0400) and *EFNA5* (*p* = 0.0228).

The RT-qPCR results indicated that the downregulated genes were *CSMD2, CREBBP, ATXN1,* and *B3GNT2* (Figure 4B). From those genes downregulated, expression was the highest for *CSMD2* with a 2-fold (*p* = 0.0022), *ATNX1* with a 1-fold (*p* = 0.0043), and *CREBBP* with a 0.5-fold (*p* = 0.0152). These results demonstrate that the miRNAs identified in this study could impact gene expression and thus validate their associated link to HD.

## 4 Discussion

miRNAs are widely expressed in the central nervous system, and dysregulation of miRNAs has been reportedly associated with NDs such as HD (Cao et al., 2016; Dong and Cong, 2021). Recently, differential expression of miRNAs has been reported in human, animal, and cell models of HD using several methods such as microarray analysis, real-time PCR, and bioinformatic studies (Sinha et al., 2010; Spronck et al., 2019; Guo et al., 2022). In this study, we examined miRNA regulation in the human SH-SY5Y HD cell model and identified that miRNA–mRNA interaction was significantly DE in Q23 and Q74 cell lines. This is a significant study that analyzes miRNA regulation in a human model to help expand on previous results in NDs, including HD.

Using the HTG EdgeSeq protocol to analyze differential expression profiles of miRNA in Q23 and Q74 cell lines showed that 354 miRNAs (top 18 miRNAs) were DE (*p* < 0.05). qRT-PCR analysis was used to further validate gene expression and obtain miRNA targets from the MirTarget program results. As shown by the program results DE miR-3687, miR-612, miR-4261, miR-504-3p, miR-126-5p,and miR-411-5p bind to the mRNA of the B*3GNT2, CSMD2, ATN1, CREBBP, ATXN1, EMIN4,* and *EFNA5* genes, which are associated with neurodegenerative diseases (Table 2).

Expression of miR-3687 was most elevated in human cell models compared to other DE miRNAs. At present, there are only a few studies on miR-3687 in NDs. A recent report demonstrated that in the analysis of microRNA expression in motor neuron-like cells derived from human cord blood mesenchymal stem cells, miR-3687 was significantly upregulated (Sanooghi et al., 2022). Moreover, Target Scan’s findings indicate that miR-3687 is connected to DNA-binding proteins and proteins related to ubiquitin (Agarwal et al., 2015; Hagio et al., 2019). Ubiquitin-binding proteins are considered a major component of neurotoxic protein aggregates that characterize many neurodegenerative diseases (Schmidt et al., 2021). Dysfunction of the ubiquitin-proteasome system is the main factor that initiates and aggravates the pathogenesis of neurodegenerative diseases. For example, some studies informed that CHIP ubiquitin ligase could suppress the aggregation of the mutant Htt fragment (Miller et al., 2005). Our results show that miR-3687 binds to the mRNA of the *B3GNT2* gene, which is associated with autoimmune diseases. Among the DE genes associated with immunity, as previously mentioned above, *B3GNT2* is involved in autoimmune disorders previously associated with the risk of schizophrenia (rheumatoid arthritis and Graves’ disease) (Sanders et al., 2013). RT-qPCR analysis showed a statistically significant (*p* < 0.01) decrease in *B3GNT2* expression in the Q74 samples compared to Q23. It could be hypothesized that differentially upregulated expression of miR-3687 could control the *B3GNT2* target gene in NDs.

miR-4261 was upregulated with *ATN1* and *CREBBP* target genes were DE (*p* < 0.05) in our study (Figures 4A,B). At present, there are only a few studies on miR-4261 and they have mainly been related to cancer (Jiao et al., 2017). However, miR-4261 levels in NDs have not previously been reported. According to the human miRNA tissue atlas, miR-4261 expression is higher in the brain expression:148.46 (reads per kilobase million) compared to other tissues (Ludwig et al., 2016). The associations of miR-4261 with candidate *ATN1* and *CREBBP* target genes play an important role in NDs. For instance, *ATN1* is thought to play a role as a nuclear transcriptional regulator important for the development of the brain and other organ systems (Palmer et al., 2019). Moreover, the function of *ATN1* may also be affected in HD, as this gene is also a cause of Poly-Q diseases. Previous studies also showed that, compared to all Poly-Q genes, *ATN1* had the highest expression across the brain (Keo et al., 2017). The *CREBBP* gene influences CREB-binding proteins that play a role in regulating the activity of various genes in tissues throughout the body (Cardinaux et al., 2000; Gao et al., 2020). Previous studies have shown that the CREBBP-binding proteins are involved in brain development and long-term memory formation (Chatterjee et al., 2020; Wang et al., 2022). *CREBBP* gene may play a role in the pathogenesis of various neurodegenerative diseases, such as HD (Steffan et al., 2000). The associations of miR-4261 to *ATN1* and *CREBBP* target genes can serve as markers for Poly-Q disorders, including HD diagnosis.

Furthermore, our results showed that miR-612 and miR-504-3p expression levels were significantly increased in Q74 in comparison with normal cell lines. miR-612 in NDs and its associated mechanism remain unclear. However, the *CSMD2* gene, which was a target of miR-612, is described as a regulator of complement activation and inflammation in the developing central nervous system (Athanasiu et al., 2017). The *CSMD* gene family has been reported to be involved in the pathogenesis of schizophrenia (Håvik et al., 2011). Chen D. et al. (2022) also found that miR-504-3p was differentially upregulated in hTau mice. We found that miR-504-3p can target the *ATXN1* gene, which has been extensively studied in NDs (Kerkhof et al., 2023). Statistical analysis of relative expression results shows that *ATXN1* gene expression was significantly downregulated (*p* < 0.01) in Q74 (Figure 4B). It is well-known that the loss of *ATXN1* results in transcriptional changes that are potentially pathogenic in neurodegenerative disease caused by the expansion of a CAG repeat (Hu and Corey, 2020).

Our results demonstrate that miR-126-5p and miR-411-5p show differentially downregulated levels in the HD model. Previous studies also showed that miR126-5p downregulation facilitates axon degeneration and neuromuscular junctions’ disruption via a non-cell autonomous mechanism in amyotrophic lateral sclerosis (Maimon et al., 2018). In addition, some studies suggest that miR-126-5p can play an important role in regulating the proliferation of endothelial cells (Esser et al., 2017). Patrício et al. (2020) demonstrated that the effects of chronic mild stress and fluoxetine treatment on miRNA levels varied across brain regions, and miR-411-5p was significantly decreased in the blood of fluoxetine-treated rats. These downregulated miR-126-5p and miR-411-5p expressions may explain and be proven to be a good measure both in animal and human studies. We determined *GEMIN4* and *EFNA5* as direct target gene of miR-126-5p and miR-411-5p using the MirTarget program. These genes were significantly upregulated in the Q74 cell lines. Overexpression of *GEMIN4* is predicted to result in nucleoplasmic accumulation of the survival motor neuron and all other tested members of the complex (Meier et al., 2018). *GEMIN4* was originally identified as a member of the survival motor neuron complex (Charroux et al., 2000). *EFNA5* gene has been associated with Alzheimer’s disease and Parkinson’s disease (Lesnick et al., 2007; Potkin et al., 2009). *EFNA5,* which is a member of the Ephrin (Eph) superfamily implicated in mediating developmental events in the nervous system, is highly expressed in the human brain and hippocampus (Martínez and Soriano, 2005; Arvanitis and Davy, 2008; Potkin et al., 2009).

The profiling of miRNA expression in the SH-SY5Y HD cell model offers a valuable understanding of the molecular alterations linked to HD pathology. There was a significant overlap in target genes which are associated with NDs and DE miRNAs. Overall, our results suggest that DE 354 miRNAs, including miR-4261, miR-126-5p, miR-411-5p, miR-612, miR-504-3p, and miR-3687, and mRNA of the *ATN1, GEMIN4, EFNA5, CSMD2, CREBBP, ATXN1,* and *B3GNT2* genes may play a pivotal role in NDs, including HD pathogenesis.

## 5 Conclusion

In conclusion, we report significant DE 354 miRNA profiles and their target genes in human cell line models. These profiles could act as early diagnosis biomarkers for pathological research of HD and other NDs. Furthermore, dysregulation of the miRNAs (miR-3687, miR-612, miR-4261, miR-504-3p, miR-126-5p, and miR-411-5p) with mRNAs (*B3GNT2, CSMD2, ATN1, CREBBP, ATXN1, EMIN4,* and *EFNA5*) identified in this study may play a decisive role in the development of diseases associated with the expansion of nucleotide repeats. Further studies should confirm the discovered functional relationships by revealing the mechanisms of pathological processes, which will serve as the basis for the development of new diagnostic strategies and alternative therapies. DE miRNAs in model cell lines can be considered potential biomarkers (early diagnosis, prognosis, and treatment monitoring) of HD and other neurodegenerative diseases. Further research in cohorts of patients and healthy controls is needed to develop such biomarkers.

## Data availability statement

The datasets presented in this study can be found in online repositories. The names of the repository/repositories and accession number(s) can be found in the article/Supplementary material.

## Ethics statement

Ethical approval was not required for the studies on humans in accordance with the local legislation and institutional requirements because only commercially available established cell lines were used. Ethical approval was not required for the studies on animals in accordance with the local legislation and institutional requirements because only commercially available established cell lines were used.

## Author contributions

AB: Conceptualization, Data curation, Formal analysis, Funding acquisition, Investigation, Methodology, Project administration, Software, Validation, Visualization, Writing – original draft, Writing – review & editing. RN: Conceptualization, Writing – review & editing. MK: Conceptualization, Writing – review & editing. AI: Conceptualization, Writing – review & editing, Data curation, Formal analysis, Investigation, Methodology, Visualization. KS: Conceptualization, Writing – review & editing. CW: Conceptualization, Writing – review & editing, Data curation, Formal analysis, Funding acquisition, Investigation, Methodology, Project administration, Resources, Software, Supervision, Validation, Visualization, Writing – original draft.

## Footnotes

^1^http://mirbase.org

^2^http://www.ncbi.nlm.nih.gov

^3^https://www.ncbi.nlm.nih.gov/tools/primer-blast/

^4^https://reveal.htgmolecular.com/

^5^https://diseases.jensenlab.org/About

^6^https://pubmed.ncbi.nlm.nih.gov/
