# Supplementary material for: Differential microRNA expression in the SH-SY5Y human cell model as potential biomarkers for Huntington’s disease
Source: Front Cell Neurosci. 2024 Jul 10;18:1399742. doi: 10.3389/fncel.2024.1399742 (PMC11267620; doi:10.3389/fncel.2024.1399742)
Supplement: Supplementary file 1 [file Table_1.DOCX]

**Profiling microRNA expression in SH-SY5Y human cell model as a biomarker for Huntington’s disease**

**Table S1.** List of oligonucleotides used in this study

| Gene | Forward Sequence (5′-3′) | Reverse Sequence (5′-3′) |
| --- | --- | --- |
| *B3GNT2* | CAACAAGTGCCGGAGGCTA | ATATCTTGTCCGGGAGCAGC |
| *CSMD2* | AACACTGTCTGTCTACGGGG | AGTGGAGCTTGGTCTGAGTG |
| *GNAO1* | GGGGCTGGAGAATCAGGAAAA | GCGTCAGCCTTTCTCTCCTT |
| *ATXN2* | AGCCAAGACATATAGAGCAGGT | AACTGCTGAGGACTGTAGGC |
| *DUSP15* | GGGCAATGGCATGACCAAG | AGCTGATCCAGGTCTTTGGC |
| *EPN3* | CCCCTAGTTCGCTCATGTCC | CCCGTCTTGAGCAGGTAGTC |
| *GLIS1* | GACTACGGTCCATCCAAGGC | GTCCAGCAGGCTCTTCTCTG |
| *CREBBP* | CTCAGAGCCAGTTTCTGCCA | GGTTAGGAAGAGCAGCACCA |
| *ATN1* | CCTGCACCAGCAAGATGCTA | TAGGGGATCCGGGTAAGGTG |
| *HAP1* | GATGCAGCTCCAGGAAGAGG | ACCTGCTCTCGATCCTCACT |
| *ATXN1* | GATCGACTCCAGCACCGTAG | GATGACCAGCCCTGTCCAAA |
| *DNM1L* | AATCGTCGTAGTGGGAACGC | TCTGCTTCCACCCCATTTTCT |
| *GEMIN4* | TTGCTGGCTTATGTGGAGGG | AACTTGTGGGTGCCGAGATT |
| *NOS3* | CCGGAAGGCTTTTGATCCCC | AACTCTTGTGCTGTTCCGGC |
| *EFNA5* | GTTGACGCTGGTGTTTCTGG | TGGTAGTCACCCCTCTGGAA |
| *CNPY1* | TGGACGAGATAGAGCACGAC | TCCTTCGTCACAGGGTCTTC |
| *KIAA1324L* | GGGTTTCCGAGCAGCCTTAT | TTTGGCTTGGTGGAACTGGG |
| *ATXN10* | CGTGAGAGCAGAAGGTGACA | CACTGGGTCAGAAAGGGGTT |
| *18S* | GTGGAGCGATTTGTCTG GTT | AACGCCACTTGTCCCTCTAA |

**Table S2.** Quality control of wild and mutant type of HD samples

| Sample Name | Percent POS | QC0 Status | Total Counts | QC1 Status | RSD | QC2 Status | QC Status | Group |
| --- | --- | --- | --- | --- | --- | --- | --- | --- |
| 377-A046139 | 0.19 | PASS | 785965 | PASS | 0.803 | PASS | PASS | control |
| 377-A046140 | 0.31 | PASS | 714911 | PASS | 0.295 | PASS | PASS | control |
| 377-A046141 | 0.28 | PASS | 733547 | PASS | 0.671 | PASS | PASS | control |
| 377-A046142 | 0.32 | PASS | 766019 | PASS | 0.65 | PASS | PASS | control |
| 377-A046145 | 0.27 | PASS | 689284 | PASS | 0.656 | PASS | PASS | disease |
| 377-A046146 | 0.37 | PASS | 696576 | PASS | 0.645 | PASS | PASS | disease |
| 377-A046147 | 0.34 | PASS | 105697 | PASS | 0.459 | PASS | PASS | disease |
| 377-A046148 | 0.41 | PASS | 820913 | PASS | 0.698 | PASS | PASS | disease |

POS - positive control probes; QC - Quality Control; RSD – Relative standard deviation

**Table S3.** Differentially expressed miRNAs between control (HTT-Q23) and mutant (HTT-Q74) groups (*p* < 0.05)

| № | miRNA | Mean expression | Fold Change disease.vs.control | adjP disease.vs.control | № | miRNA | Mean expression | Fold Change disease.vs.control | adjP disease.vs.control |
| --- | --- | --- | --- | --- | --- | --- | --- | --- | --- |
| 1 | miR-3687 | 14,63 | 3,96 | 2,72E-09 | 178 | miR-10b-5p | 10,30 | -3,61 | 1,98E-02 |
| 2 | miR-4417 | 12,23 | 2,71 | 4,03E-06 | 179 | miR-6835-5p | 6,25 | 2,33 | 2,02E-02 |
| 3 | miR-411-5p | 4,61 | -17,69 | 7,03E-04 | 180 | miR-34b-5p | 6,32 | -2,92 | 2,04E-02 |
| 4 | miR-32-5p | 8,46 | -11,61 | 8,24E-04 | 181 | miR-191-5p | 12,00 | -3,68 | 2,05E-02 |
| 5 | miR-1273g-5p | 8,92 | 2,82 | 8,24E-04 | 182 | miR-6740-5p | 6,47 | 2,34 | 2,11E-02 |
| 6 | miR-3648 | 8,36 | 2,30 | 8,24E-04 | 183 | miR-4695-5p | 9,70 | 2,17 | 2,12E-02 |
| 7 | miR-301a-3p | 10,57 | -12,57 | 8,24E-04 | 184 | miR-624-5p | 3,58 | -6,23 | 2,13E-02 |
| 8 | miR-135a-5p | 4,80 | -8,08 | 1,10E-03 | 185 | miR-181b-5p | 10,52 | -3,77 | 2,14E-02 |
| 9 | miR-612 | 7,46 | 3,14 | 1,10E-03 | 186 | miR-323b-3p | 4,04 | -4,37 | 2,16E-02 |
| 10 | miR-876-3p | 6,19 | -8,05 | 1,20E-03 | 187 | miR-7851-3p | 8,93 | 2,27 | 2,17E-02 |
| 11 | miR-889-3p | 4,18 | -14,05 | 1,30E-03 | 188 | miR-3605-5p | 6,65 | 2,14 | 2,17E-02 |
| 12 | miR-148a-3p | 9,05 | -11,07 | 1,30E-03 | 189 | miR-518b | 2,29 | -23,11 | 2,17E-02 |
| 13 | miR-126-5p | 7,88 | -11,79 | 1,40E-03 | 190 | miR-181d-5p | 10,17 | -3,87 | 2,17E-02 |
| 14 | miR-495-3p | 7,28 | -9,39 | 1,40E-03 | 191 | miR-148b-3p | 9,74 | -4,04 | 2,17E-02 |
| 15 | let-7f-2-3p | 4,43 | -7,08 | 1,40E-03 | 192 | miR-543 | 6,94 | -2,87 | 2,18E-02 |
| 16 | miR-30b-5p | 11,11 | -9,39 | 1,50E-03 | 193 | miR-4725-3p | 7,71 | 2,72 | 2,19E-02 |
| 17 | miR-23c | 8,58 | -9,67 | 1,50E-03 | 194 | miR-29a-3p | 10,50 | -3,53 | 2,19E-02 |
| 18 | miR-410-3p | 5,47 | -6,92 | 1,70E-03 | 195 | miR-4319 | 3,25 | -7,51 | 2,22E-02 |
| 19 | miR-1273h-5p | 12,03 | 2,22 | 1,70E-03 | 196 | miR-5192 | 6,58 | 2,19 | 2,22E-02 |
| 20 | miR-379-5p | 5,23 | -8,68 | 1,80E-03 | 197 | miR-30a-5p | 11,28 | -3,57 | 2,29E-02 |
| 21 | miR-369-3p | 4,35 | -10,37 | 1,90E-03 | 198 | miR-1224-5p | 7,44 | 1,86 | 2,32E-02 |
| 22 | miR-377-3p | 7,04 | -8,21 | 1,90E-03 | 199 | miR-204-3p | 10,46 | 3,11 | 2,33E-02 |
| 23 | miR-34a-3p | 6,88 | -5,43 | 2,00E-03 | 200 | miR-376a-5p | 5,39 | -3,67 | 2,37E-02 |
| 24 | miR-655-3p | 4,07 | -14,72 | 2,00E-03 | 201 | miR-6799-5p | 9,77 | 1,91 | 2,43E-02 |
| 25 | miR-101-3p | 10,85 | -7,66 | 2,10E-03 | 202 | miR-548b-3p | 5,01 | -3,22 | 2,43E-02 |
| 26 | miR-660-5p | 8,14 | -8,06 | 2,10E-03 | 203 | miR-539-3p | 2,28 | -14,05 | 2,43E-02 |
| 27 | let-7d-5p | 10,90 | -10,28 | 2,20E-03 | 204 | miR-6894-5p | 10,40 | 2,23 | 2,45E-02 |
| 28 | miR-380-3p | 3,53 | -16,12 | 2,60E-03 | 205 | miR-455-5p | 3,58 | -4,50 | 2,46E-02 |
| 29 | miR-651-5p | 3,43 | -7,82 | 2,60E-03 | 206 | miR-6516-5p | 11,13 | -2,25 | 2,46E-02 |
| 30 | miR-188-3p | 3,80 | -6,43 | 2,80E-03 | 207 | miR-1305 | 3,10 | -5,37 | 2,50E-02 |
| 31 | miR-4484 | 8,53 | 2,04 | 2,80E-03 | 208 | miR-6165 | 10,10 | 1,94 | 2,50E-02 |
| 32 | miR-153-3p | 10,38 | -7,39 | 2,80E-03 | 209 | miR-100-5p | 10,06 | -3,39 | 2,51E-02 |
| 33 | let-7a-3p | 5,63 | -6,97 | 2,80E-03 | 210 | miR-4800-5p | 8,43 | 2,31 | 2,51E-02 |
| 34 | miR-582-5p | 6,40 | -6,63 | 2,80E-03 | 211 | miR-4696 | 2,24 | -15,94 | 2,54E-02 |
| 35 | miR-20b-5p | 12,18 | -7,94 | 2,80E-03 | 212 | miR-5187-3p | 2,66 | -16,71 | 2,60E-02 |
| 36 | miR-137 | 11,52 | -8,64 | 2,80E-03 | 213 | miR-708-5p | 11,33 | -3,46 | 2,63E-02 |
| 37 | miR-301b | 10,03 | -6,00 | 2,80E-03 | 214 | miR-3909 | 3,46 | -4,67 | 2,68E-02 |
| 38 | miR-340-3p | 5,30 | -8,43 | 2,80E-03 | 215 | miR-3652 | 7,96 | 1,91 | 2,68E-02 |
| 39 | miR-340-5p | 7,92 | -7,97 | 2,90E-03 | 216 | miR-6830-5p | 7,63 | 2,11 | 2,70E-02 |
| 40 | miR-542-3p | 4,97 | -6,36 | 3,50E-03 | 217 | miR-4772-5p | 2,60 | -21,01 | 2,74E-02 |
| 41 | miR-32-3p | 3,10 | -32,83 | 3,60E-03 | 218 | miR-18a-5p | 11,04 | -3,19 | 2,74E-02 |
| 42 | miR-5088-5p | 7,02 | 2,57 | 3,60E-03 | 219 | miR-22-5p | 7,44 | -2,87 | 2,74E-02 |
| 43 | miR-196a-3p | 4,39 | -6,60 | 3,90E-03 | 220 | miR-6748-5p | 6,06 | 2,30 | 2,74E-02 |
| 44 | miR-363-3p | 9,38 | -5,97 | 3,90E-03 | 221 | miR-222-3p | 6,16 | -2,88 | 2,74E-02 |
| 45 | miR-4309 | 5,35 | -2,83 | 4,10E-03 | 222 | miR-4428 | 7,30 | 2,16 | 2,74E-02 |
| 46 | miR-20a-5p | 12,44 | -6,16 | 4,10E-03 | 223 | miR-4726-5p | 8,06 | 2,25 | 2,75E-02 |
| 47 | miR-154-3p | 5,32 | -7,43 | 4,10E-03 | 224 | miR-6769a-5p | 8,21 | 2,45 | 2,76E-02 |
| 48 | miR-30c-5p | 11,78 | -6,04 | 4,10E-03 | 225 | miR-6503-3p | 6,63 | 1,82 | 2,79E-02 |
| 49 | miR-29b-3p | 10,08 | -6,91 | 4,10E-03 | 226 | miR-4646-5p | 9,32 | 2,43 | 2,79E-02 |
| 50 | miR-3154 | 7,99 | 2,44 | 4,20E-03 | 227 | miR-146b-5p | 7,14 | -3,00 | 2,79E-02 |
| 51 | miR-10a-5p | 6,60 | -5,01 | 4,30E-03 | 228 | miR-2467-3p | 9,00 | 2,00 | 2,79E-02 |
| 52 | miR-27a-3p | 12,96 | -7,76 | 4,30E-03 | 229 | miR-106b-5p | 13,43 | -3,34 | 2,79E-02 |
| 53 | miR-656-3p | 3,25 | -14,16 | 4,30E-03 | 230 | miR-5584-5p | 6,60 | 2,03 | 2,79E-02 |
| 54 | miR-1273c | 9,80 | 2,00 | 4,30E-03 | 231 | miR-1207-5p | 10,69 | 2,03 | 2,79E-02 |
| 55 | miR-196a-5p | 7,25 | -7,11 | 4,30E-03 | 232 | miR-3157-5p | 5,61 | -2,04 | 2,80E-02 |
| 56 | miR-338-3p | 9,65 | -7,18 | 4,30E-03 | 233 | miR-4444 | 9,94 | 1,82 | 2,80E-02 |
| 57 | miR-152-3p | 8,82 | -7,70 | 4,30E-03 | 234 | miR-6719-3p | 2,68 | -12,75 | 2,87E-02 |
| 58 | miR-190a-5p | 3,25 | -18,37 | 4,30E-03 | 235 | miR-3606-3p | 2,43 | -12,72 | 2,87E-02 |
| 59 | miR-299-5p | 4,98 | -4,15 | 4,40E-03 | 236 | miR-6716-5p | 7,94 | 2,24 | 2,89E-02 |
| 60 | miR-126-3p | 8,95 | -7,10 | 4,60E-03 | 237 | miR-6124 | 10,57 | 2,07 | 2,90E-02 |
| 61 | miR-545-3p | 4,98 | -6,79 | 4,70E-03 | 238 | miR-361-5p | 10,69 | -3,84 | 2,92E-02 |
| 62 | miR-4758-5p | 7,55 | 2,06 | 4,70E-03 | 239 | miR-5705 | 2,84 | -12,11 | 2,94E-02 |
| 63 | miR-362-3p | 7,65 | -6,10 | 5,00E-03 | 240 | miR-3064-5p | 9,74 | 2,27 | 2,95E-02 |
| 64 | miR-342-3p | 10,23 | -6,62 | 5,20E-03 | 241 | miR-4685-5p | 6,66 | 2,02 | 2,98E-02 |
| 65 | miR-107 | 12,16 | -6,73 | 5,30E-03 | 242 | miR-99a-5p | 10,46 | -3,28 | 2,98E-02 |
| 66 | miR-6779-5p | 7,00 | 2,26 | 5,30E-03 | 243 | miR-3158-5p | 7,17 | 2,01 | 3,00E-02 |
| 67 | miR-590-5p | 8,76 | -6,65 | 5,30E-03 | 244 | miR-4419b | 8,58 | 1,78 | 3,04E-02 |
| 68 | miR-4257 | 7,04 | 2,55 | 5,40E-03 | 245 | miR-6833-5p | 7,60 | 2,20 | 3,04E-02 |
| 69 | miR-95-3p | 7,28 | -5,94 | 5,50E-03 | 246 | miR-765 | 9,77 | 2,16 | 3,04E-02 |
| 70 | miR-27b-3p | 12,54 | -6,46 | 5,50E-03 | 247 | miR-26b-3p | 4,74 | -2,55 | 3,04E-02 |
| 71 | miR-376b-3p | 6,64 | -5,38 | 5,50E-03 | 248 | miR-6760-5p | 9,10 | 2,21 | 3,04E-02 |
| 72 | miR-921 | 6,91 | 2,16 | 5,50E-03 | 249 | miR-7108-5p | 8,44 | 1,87 | 3,04E-02 |
| 73 | miR-199b-5p | 10,38 | -5,49 | 5,60E-03 | 250 | miR-4305 | 2,45 | -11,76 | 3,08E-02 |
| 74 | miR-96-5p | 8,29 | -5,87 | 5,60E-03 | 251 | miR-1273e | 10,63 | 1,83 | 3,10E-02 |
| 75 | miR-103a-3p | 12,36 | -6,48 | 5,60E-03 | 252 | miR-4701-3p | 5,61 | 2,30 | 3,19E-02 |
| 76 | miR-132-3p | 8,56 | -5,62 | 5,60E-03 | 253 | miR-542-5p | 4,54 | -4,24 | 3,21E-02 |
| 77 | miR-649 | 7,91 | 2,20 | 6,00E-03 | 254 | miR-3133 | 2,15 | -20,86 | 3,23E-02 |
| 78 | miR-142-5p | 3,16 | -11,81 | 6,10E-03 | 255 | miR-6778-5p | 11,43 | 2,37 | 3,31E-02 |
| 79 | miR-4496 | 10,33 | 2,40 | 6,10E-03 | 256 | miR-2392 | 8,08 | 1,91 | 3,34E-02 |
| 80 | miR-4789-3p | 2,62 | -29,30 | 6,30E-03 | 257 | miR-711 | 7,64 | 2,04 | 3,35E-02 |
| 81 | miR-323a-3p | 5,55 | -5,97 | 6,60E-03 | 258 | miR-633 | 1,77 | -22,50 | 3,36E-02 |
| 82 | miR-3934-5p | 8,57 | 2,14 | 6,60E-03 | 259 | miR-641 | 3,20 | -4,18 | 3,42E-02 |
| 83 | miR-17-5p | 13,17 | -5,31 | 6,60E-03 | 260 | miR-8085 | 7,54 | 2,35 | 3,42E-02 |
| 84 | miR-663b | 10,55 | 2,02 | 6,60E-03 | 261 | miR-4487 | 7,31 | 2,18 | 3,42E-02 |
| 85 | miR-382-3p | 3,98 | -8,28 | 6,60E-03 | 262 | miR-1306-3p | 6,23 | 1,94 | 3,44E-02 |
| 86 | miR-9-3p | 9,80 | -5,27 | 6,80E-03 | 263 | miR-4693-5p | 2,15 | -16,40 | 3,44E-02 |
| 87 | miR-9-5p | 12,15 | -5,41 | 6,90E-03 | 264 | miR-4436b-3p | 8,75 | 1,81 | 3,44E-02 |
| 88 | miR-4499 | 7,56 | 2,39 | 7,20E-03 | 265 | miR-4481 | 7,20 | 1,99 | 3,44E-02 |
| 89 | miR-23b-3p | 13,07 | -5,81 | 7,20E-03 | 266 | miR-6806-3p | 2,68 | -7,12 | 3,44E-02 |
| 90 | miR-29c-3p | 10,14 | -4,37 | 7,40E-03 | 267 | miR-6890-5p | 6,84 | 2,03 | 3,44E-02 |
| 91 | miR-487a-3p | 4,27 | -8,47 | 7,60E-03 | 268 | miR-24-3p | 13,43 | -3,23 | 3,46E-02 |
| 92 | miR-3925-5p | 6,72 | 2,18 | 7,60E-03 | 269 | miR-218-5p | 14,13 | -3,07 | 3,47E-02 |
| 93 | miR-23a-3p | 13,16 | -5,42 | 7,60E-03 | 270 | miR-6739-5p | 5,95 | 2,17 | 3,47E-02 |
| 94 | miR-302b-3p | 2,01 | -23,84 | 7,60E-03 | 271 | miR-140-5p | 9,09 | -3,01 | 3,47E-02 |
| 95 | miR-3117-5p | 3,75 | -6,25 | 7,70E-03 | 272 | miR-367-3p | 2,27 | -12,58 | 3,47E-02 |
| 96 | miR-508-5p | 2,19 | -21,54 | 7,90E-03 | 273 | miR-6796-5p | 5,21 | 2,20 | 3,51E-02 |
| 97 | miR-6726-5p | 7,69 | 2,06 | 7,90E-03 | 274 | miR-411-3p | 3,32 | -7,41 | 3,51E-02 |
| 98 | miR-3607-5p | 11,95 | -4,77 | 7,90E-03 | 275 | miR-6848-5p | 7,28 | 1,94 | 3,51E-02 |
| 99 | miR-374b-5p | 10,26 | -7,37 | 7,90E-03 | 276 | miR-6511a-5p | 8,45 | 1,75 | 3,55E-02 |
| 100 | miR-196b-5p | 3,23 | -11,53 | 8,90E-03 | 277 | miR-4687-3p | 6,99 | 1,91 | 3,59E-02 |
| 101 | miR-106a-5p | 11,84 | -4,35 | 8,90E-03 | 278 | miR-365a-3p | 8,40 | -3,08 | 3,59E-02 |
| 102 | miR-374a-3p | 1,87 | -22,09 | 8,90E-03 | 279 | miR-888-3p | 2,12 | -25,59 | 3,72E-02 |
| 103 | miR-33a-3p | 5,15 | -6,39 | 9,20E-03 | 280 | miR-30d-5p | 11,52 | -3,23 | 3,73E-02 |
| 104 | miR-21-5p | 13,26 | -4,88 | 9,20E-03 | 281 | miR-1911-5p | 3,14 | -11,09 | 3,73E-02 |
| 105 | miR-205-5p | 3,39 | -8,63 | 9,50E-03 | 282 | miR-551b-3p | 8,59 | -2,94 | 3,73E-02 |
| 106 | miR-3198 | 6,89 | 2,43 | 9,50E-03 | 283 | miR-3156-5p | 5,94 | 1,98 | 3,73E-02 |
| 107 | miR-6859-5p | 7,09 | 2,22 | 9,50E-03 | 284 | miR-4261 | 7,60 | 2,10 | 3,74E-02 |
| 108 | miR-329-3p | 6,75 | -3,46 | 9,80E-03 | 285 | miR-335-5p | 3,59 | -5,06 | 3,75E-02 |
| 109 | miR-1299 | 8,02 | 2,22 | 9,80E-03 | 286 | miR-4498 | 6,22 | 1,84 | 3,79E-02 |
| 110 | miR-577 | 2,46 | -12,91 | 9,80E-03 | 287 | miR-105-5p | 6,97 | -3,25 | 3,84E-02 |
| 111 | miR-511-3p | 1,85 | -41,65 | 1,04E-02 | 288 | miR-380-5p | 2,22 | -13,03 | 3,84E-02 |
| 112 | miR-381-3p | 5,83 | -4,11 | 1,04E-02 | 289 | miR-1185-1-3p | 3,76 | -4,53 | 3,87E-02 |
| 113 | miR-4705 | 4,79 | -4,67 | 1,05E-02 | 290 | miR-892a | 2,32 | -17,32 | 3,90E-02 |
| 114 | miR-6829-5p | 7,04 | 2,45 | 1,08E-02 | 291 | miR-455-3p | 4,69 | -3,21 | 3,90E-02 |
| 115 | miR-489-3p | 4,65 | -6,61 | 1,09E-02 | 292 | miR-3611 | 2,80 | -9,36 | 3,91E-02 |
| 116 | miR-4442 | 8,00 | 1,96 | 1,12E-02 | 293 | miR-20a-3p | 9,03 | -2,86 | 3,91E-02 |
| 117 | let-7i-5p | 10,55 | -4,62 | 1,12E-02 | 294 | miR-5703 | 7,29 | 1,81 | 3,91E-02 |
| 118 | miR-15b-3p | 7,69 | -4,55 | 1,14E-02 | 295 | miR-6746-5p | 9,68 | 2,02 | 3,91E-02 |
| 119 | miR-130a-3p | 11,91 | -4,25 | 1,14E-02 | 296 | miR-15b-5p | 12,92 | -3,25 | 3,96E-02 |
| 120 | miR-1185-2-3p | 3,97 | -4,89 | 1,18E-02 | 297 | miR-144-5p | 1,89 | -17,37 | 3,96E-02 |
| 121 | miR-595 | 5,09 | 2,71 | 1,20E-02 | 298 | miR-182-3p | 4,07 | -2,56 | 4,03E-02 |
| 122 | miR-504-3p | 7,92 | 2,30 | 1,20E-02 | 299 | miR-1251-3p | 2,67 | -9,86 | 4,04E-02 |
| 123 | miR-19b-3p | 14,31 | -3,85 | 1,27E-02 | 300 | miR-125b-2-3p | 3,19 | -4,35 | 4,08E-02 |
| 124 | miR-6791-5p | 7,69 | 1,98 | 1,27E-02 | 301 | miR-1182 | 6,46 | 1,98 | 4,08E-02 |
| 125 | miR-5684 | 8,11 | 2,09 | 1,29E-02 | 302 | miR-20b-3p | 4,98 | -2,93 | 4,14E-02 |
| 126 | miR-153-5p | 4,20 | -4,50 | 1,29E-02 | 303 | miR-6827-5p | 6,68 | 2,12 | 4,16E-02 |
| 127 | miR-18b-5p | 9,74 | -3,64 | 1,29E-02 | 304 | miR-1273d | 12,21 | 1,83 | 4,20E-02 |
| 128 | miR-376a-3p | 8,02 | -3,47 | 1,34E-02 | 305 | miR-6780b-3p | 3,33 | -4,56 | 4,22E-02 |
| 129 | miR-4522 | 4,43 | -5,42 | 1,34E-02 | 306 | miR-144-3p | 2,14 | -13,00 | 4,27E-02 |
| 130 | miR-409-5p | 4,98 | -2,70 | 1,34E-02 | 307 | miR-7515 | 6,48 | 2,32 | 4,29E-02 |
| 131 | miR-218-1-3p | 4,36 | -4,13 | 1,35E-02 | 308 | miR-4778-3p | 1,87 | -15,63 | 4,37E-02 |
| 132 | miR-616-3p | 6,04 | 2,14 | 1,36E-02 | 309 | miR-365b-5p | 8,04 | 2,47 | 4,37E-02 |
| 133 | miR-6884-5p | 6,06 | 2,67 | 1,37E-02 | 310 | miR-450a-1-3p | 2,73 | -4,11 | 4,49E-02 |
| 134 | miR-454-5p | 4,89 | -4,61 | 1,46E-02 | 311 | miR-30e-3p | 8,12 | -3,11 | 4,53E-02 |
| 135 | miR-216a-5p | 2,87 | -16,79 | 1,46E-02 | 312 | let-7d-3p | 5,62 | -3,33 | 4,53E-02 |
| 136 | miR-4725-5p | 7,08 | 2,25 | 1,46E-02 | 313 | miR-7846-3p | 7,08 | 1,95 | 4,56E-02 |
| 137 | miR-6862-5p | 6,15 | 2,49 | 1,46E-02 | 314 | miR-6817-3p | 2,33 | -8,50 | 4,59E-02 |
| 138 | miR-199a-5p | 12,48 | -4,02 | 1,48E-02 | 315 | miR-4664-3p | 4,87 | -2,46 | 4,59E-02 |
| 139 | miR-7106-5p | 7,47 | 1,92 | 1,49E-02 | 316 | miR-6880-5p | 10,27 | 2,23 | 4,59E-02 |
| 140 | miR-5585-3p | 11,32 | 1,98 | 1,51E-02 | 317 | miR-892c-3p | 2,21 | -21,69 | 4,61E-02 |
| 141 | miR-4430 | 8,68 | 2,59 | 1,51E-02 | 318 | miR-6776-5p | 6,22 | 1,98 | 4,61E-02 |
| 142 | miR-19a-5p | 5,04 | -3,34 | 1,51E-02 | 319 | miR-1229-5p | 7,89 | 1,82 | 4,63E-02 |
| 143 | miR-654-3p | 6,06 | -2,78 | 1,51E-02 | 320 | miR-1587 | 6,35 | 1,88 | 4,66E-02 |
| 144 | miR-592 | 6,29 | -4,42 | 1,51E-02 | 321 | miR-374c-5p | 7,06 | -2,44 | 4,66E-02 |
| 145 | miR-6516-3p | 9,59 | -2,51 | 1,55E-02 | 322 | miR-628-5p | 4,19 | -3,57 | 4,67E-02 |
| 146 | miR-19a-3p | 13,00 | -3,65 | 1,55E-02 | 323 | miR-183-5p | 7,33 | -2,99 | 4,67E-02 |
| 147 | miR-182-5p | 8,11 | -4,63 | 1,58E-02 | 324 | miR-1185-5p | 3,92 | -2,84 | 4,67E-02 |
| 148 | miR-1179 | 3,94 | -5,72 | 1,60E-02 | 325 | miR-515-5p | 2,44 | -12,17 | 4,68E-02 |
| 149 | miR-376c-3p | 8,63 | -4,07 | 1,62E-02 | 326 | miR-587 | 2,41 | -15,54 | 4,69E-02 |
| 150 | miR-6856-5p | 6,60 | 2,11 | 1,64E-02 | 327 | miR-5702 | 1,88 | -23,31 | 4,69E-02 |
| 151 | miR-450a-5p | 5,72 | -3,82 | 1,64E-02 | 328 | miR-6775-5p | 10,26 | 1,80 | 4,69E-02 |
| 152 | miR-384 | 2,28 | -28,56 | 1,64E-02 | 329 | miR-5584-3p | 2,11 | -7,82 | 4,69E-02 |
| 153 | miR-1 | 3,90 | -7,66 | 1,65E-02 | 330 | miR-25-3p | 13,49 | -2,77 | 4,70E-02 |
| 154 | miR-4486 | 9,17 | 2,28 | 1,65E-02 | 331 | miR-10b-3p | 5,19 | -2,17 | 4,71E-02 |
| 155 | miR-4311 | 5,71 | 2,41 | 1,65E-02 | 332 | miR-520f-3p | 1,50 | -18,48 | 4,71E-02 |
| 156 | miR-937-5p | 7,64 | 1,92 | 1,65E-02 | 333 | miR-6772-5p | 6,06 | 1,92 | 4,76E-02 |
| 157 | let-7c-5p | 12,33 | -4,90 | 1,65E-02 | 334 | miR-5093 | 7,03 | 2,39 | 4,81E-02 |
| 158 | miR-6893-5p | 7,59 | 2,12 | 1,65E-02 | 335 | miR-6757-5p | 8,04 | 2,06 | 4,81E-02 |
| 159 | miR-2114-5p | 3,50 | -18,40 | 1,65E-02 | 336 | miR-4514 | 5,77 | 2,18 | 4,83E-02 |
| 160 | miR-4419a | 7,81 | 2,38 | 1,66E-02 | 337 | miR-885-5p | 5,82 | -3,57 | 4,83E-02 |
| 161 | miR-30e-5p | 10,57 | -3,81 | 1,68E-02 | 338 | miR-660-3p | 5,36 | -2,19 | 4,83E-02 |
| 162 | miR-4270 | 9,18 | 2,19 | 1,72E-02 | 339 | miR-6715b-3p | 3,75 | -4,44 | 4,83E-02 |
| 163 | miR-494-3p | 6,59 | -2,69 | 1,72E-02 | 340 | miR-425-5p | 11,15 | -2,69 | 4,85E-02 |
| 164 | miR-192-5p | 6,34 | -3,65 | 1,72E-02 | 341 | miR-4301 | 1,87 | -14,45 | 4,85E-02 |
| 165 | miR-1273a | 8,94 | 1,76 | 1,72E-02 | 342 | miR-624-3p | 2,68 | -6,21 | 4,87E-02 |
| 166 | miR-487b-3p | 6,44 | -4,03 | 1,72E-02 | 343 | miR-4291 | 5,14 | -2,03 | 4,87E-02 |
| 167 | miR-6086 | 9,53 | 2,24 | 1,72E-02 | 344 | miR-24-2-5p | 7,42 | -2,50 | 4,87E-02 |
| 168 | let-7g-3p | 5,40 | -2,91 | 1,73E-02 | 345 | miR-200c-3p | 5,71 | -2,84 | 4,91E-02 |
| 169 | miR-6837-5p | 7,39 | 2,10 | 1,74E-02 | 346 | miR-7159-5p | 2,20 | -12,68 | 4,91E-02 |
| 170 | miR-4534 | 9,01 | 2,23 | 1,81E-02 | 347 | miR-876-5p | 7,65 | -2,20 | 4,92E-02 |
| 171 | miR-128-3p | 9,99 | -3,97 | 1,81E-02 | 348 | miR-4505 | 8,68 | 1,78 | 4,93E-02 |
| 172 | miR-4535 | 7,19 | 2,51 | 1,81E-02 | 349 | miR-296-3p | 7,26 | 1,92 | 4,93E-02 |
| 173 | miR-6807-5p | 7,03 | 2,14 | 1,82E-02 | 350 | miR-193a-3p | 6,00 | -2,02 | 4,93E-02 |
| 174 | miR-7-2-3p | 3,03 | -6,95 | 1,97E-02 | 351 | miR-7703 | 3,31 | -8,31 | 4,93E-02 |
| 175 | miR-485-3p | 5,30 | -3,99 | 1,97E-02 | 352 | miR-491-3p | 3,18 | -4,69 | 4,95E-02 |
| 176 | miR-8089 | 8,64 | 2,14 | 1,97E-02 | 353 | miR-3714 | 4,59 | -2,69 | 4,95E-02 |
| 177 | miR-4446-3p | 6,34 | 2,22 | 1,97E-02 | 354 | miR-4743-5p | 7,09 | 2,07 | 4,96E-02 |
